# Cloning and Characterization of Three *Eimeria tenella* Lipid Phosphate Phosphatases

**DOI:** 10.1371/journal.pone.0122736

**Published:** 2015-04-10

**Authors:** Aijiang Guo, Jianping Cai, Xuenong Luo, Shaohua Zhang, Junling Hou, Hui Li, Xuepeng Cai

**Affiliations:** 1 State Key Laboratory of Veterinary Etiological Biology, Key Laboratory of Veterinary Parasitology of Gansu Province, Lanzhou Veterinary Research Institute, Chinese Academy of Agricultural Sciences, Lanzhou, Gansu, China; 2 Jiangsu Co-Innovation Center for Prevention and Control of Important Animal Infectious Disease, Yangzhou, Jiangsu, China; NIAID, UNITED STATES

## Abstract

Although lipid phosphate phosphatases (LPPs) play an important role in cellular signaling in addition to lipid biosynthesis, little is thus far known about parasite LPPs. In this study, we characterized three *Eimeria tenella* cDNA clones encoding LPP named EtLPP1, EtLPP2 and EtLPP3. Key structural features previously described in LPPs, including the three conserved domains proposed as catalytic sites, a single conserved N-glycosylation site, and putative transmembrane domains were discovered in the three resulting EtLPP amino acid sequences. Expression of His6-tagged EtLPP1, -2, and -3 in HEK293 cells produced immunoreactive proteins with variable molecular sizes, suggesting the presence of multiple forms of each of the three EtLPPs. The two faster-migrating protein bands below each of the three EtLPP proteins were found to be very similar to the porcine 35-kDa LPP enzyme in their molecular size and the extent of their N-glycosylation, suggesting that the three EtLPPs are partially N-glycosylated. Kinetic analyses of the activity of the three enzymes against PA, LPA, C1P and S1P showed that Km values for each of the substrates were (in μM) 284, 46, 28, and 22 for EtLPP1; 369, 179, 237, and 52 for EtLPP2; and 355, 83, and 260 for EtLPP3. However, EtLPP3 showed negligible activity on S1P. These results confirmed that the three EtLPPs have broad substrate specificity. The results also indicated that despite structural similarities, the three EtLPPs may play distinct functions through their different models of substrate preference. Furthermore, particularly high expression levels of the three EtLPP genes were detected in the sporozoite stage of the *E*. *tenella* life cycle (p<0.001), suggesting that their encoded proteins might play an important biological function in the sporozoite stage.

## Introduction

Lipid phosphate phosphatases (LPP) (EC 3.1.3.4) catalyze the dephosphorylation of lipid phosphates, yielding their dephosphorylated counterparts and inorganic phosphate [1]. LPP enzymes have roles in both the synthesis of lipids and the decrease and /or increase of lipid-signaling molecules both in yeast and in mammalian systems [2–7]. LPP enzymes are also called type 2 phosphatidate phosphohydrolases (PAP2) and utilize the broad lipid phosphate substrates, which are characterized by having no Mg^2+^ requirement for catalytic activity [8]. LPPs are generally conserved in domain and structure. They comprise a three-domain lipid phosphate phosphatase motif consisting of the conserved sequences KXXXXXXRP (domain 1), XSGH (domain 2) and XRXXXXXHXXXD (domain 3), which is essential for enzymatic activity [9–12]. Moreover, LPP is an integral membrane protein with six putative membrane-spanning regions confined to the vacuole and Golgi membranes, with the active site predicted to be at the cell surface or on the luminal surface [11, 13]. A consensus N-glycosylation site is present between conserved domain 1 and domain 2 of LPP protein [14].

The LPPs belong to a phosphatase superfamily that includes bacterial acid phosphates [15], bacterial and yeast diacylglycerol pyrophosphatases [16, 17], yeast LPP [18, 19], fungal chloroperoxidase [20], the *Drosophila* protein Wunen [21], mammalian glucose 6-phosphatase (G-6-Pase) [22], and rat Dri42 [23]. The roles of LPP enzymes in lipid synthesis and lipid signaling have been described in mammals and yeast [2, 3]. However, little is known about the LPP superfamily in apicomplexa.

Coccidiosis, caused by various *Eimeria* species, is a major parasitic disease in chickens. With the availability of *E*. *tenella* genomic resources, full-length cDNA encoding of *E*. *tenella* LPP enzymes (EtLPP-1, -2 and -3) have been cloned in this study. Three enzymes were expressed as His6-tagged proteins by transient transfection of HEK293 cells. The activity of the three immunopurified EtLPPs enzymes against phosphatidate (PA), lysophosphatidate (LPA), sphingosine-1-phosphate (S1P) and ceramide-1-phosphate (C1P) was studied. Furthermore, expression analysis of the EtLPPs at different stages of the *E*. *tenella* life cycle was conducted to analyze their possible biological function. To our knowledge, this study is the first to attempt to identify lipid phosphate phosphatases in parasites and will provide a foundation for elucidating their biochemical regulation.

## Materials and Methods

### Cell Culture

Human embryonic kidney (HEK) 293 cells were cultured in DMEM (Life Technologies, Gaithersburg, MD, USA) supplemented with 10% fetal calf serum (Gemini, Calabasas, CA, USA) and 100 U/ml penicillin–streptomycin.

### *E*. *tenella* infection in chickens

The Lanzhou-1 strain of *E*. *tenella* was originally isolated in the field in Lanzhou, China and maintained in the Lanzhou Veterinary Research Institute. Parasite oocysts were harvested, sporulated and stored as previously described [24]. 1-day-old pathogen-free ISA Brown chickens were purchased from Xigu Farms, Lanzhou, China. All animals were handled in strict accordance with the Animal Ethics Procedures and Guidelines of the People's Republic of China, and the study was approved by the Animal Ethics Committee of Lanzhou Veterinary Research Institute, Chinese Academy of Agricultural Sciences (No. LVRIAEC2010-002). The detailed protocols for the care and use of animals and for the experimental procedures were identical to those described in our previous study [25]. The chickens were euthanized with CO_2_, and then cervically dislocated before removing tissue samples. All efforts were made to minimize suffering.

### Isolation and purification of merozoites

12-day-old chickens were infected with 1×10^5^ sporulated *E*. *tenella* oocysts. A modification of the method outlined by M.Q. Xie [26] was used to purify the merozoites. Ceca were removed at five days post infection. Second-generation merozoites of *E*. *tenella* were purified using a host tissue digestion fluid containing 0.25% trypsin and 0.5% taurodeoxycholic acid, to liberate the merozoites from the parasitized ceca.

### Recovery of oocysts of *E*. *tenella* from the ceca and collecting differentially sporulated oocysts during oocyst sporulation

Chickens were infected with 1×10^5^ sporulated *E*. *tenella* oocysts at 12-days of age and sacrificed after 7.5 additional days (19.5 days old). Oocysts were purified from the ceca of infected chickens as described elsewhere [24]. Samples of oocysts at various levels of sporulation (1.5%, 48%, and 96%) were collected. Sporozoites were prepared from 96% sporulated oocysts by shocking with glass beads (1.4mm), sporocysts were released, and then incubated with 0.25% trypsin and 0.75% taurodeoxycholic acid in PBS (pH7.6) at 42°C for 30min. Free sporozoites were washed and purified and then immediately used for isolating RNA.

### Prediction and RT-PCR amplification of *E*. *tenella* cDNAs encoding EtLPP enzymes

The potential ORF-coding LPPs of *E*. *tenella* were predicted using multiple alignments of translated protein fragments from the contigs in the *E*. *tenella* genomic database (http://www.sanger.ac.uk/projects/E-tenella/) and other apicomplexa/model organism homologue enzymes. Total RNA was extracted from sporozoites using TRIZOL (Life Technologies, Gaithersburg, MD, USA), and reverse transcription was performed using a PrimeScript RT kit (Takara Biotechnology, Dalian, China). The primers used for amplifying the complete coding regions for the putative LPP isozymes are shown in Table 1. The amplified fragments were sub-cloned into pGEM-T Easy (Promega Corporation, Madison, WI, USA) and sequenced in Shanghai Sangong Biotech Co., Ltd (Sangon, China). Transmembrane regions were predicted by Split 4.0 [27]. N-glycosylation sites were predicted using GlycoEP [28] (http://www.imtech.res.in/raghava/glycoep/index.html).

**Table 1:** Oligonucleotides used for PCR and real-time PCR.

Primers name	Oligonucleotide sequence
EtLPP3-F (Primer for ORF sequences)	5'-ATGCAGCTGGCAACGGCT-3'
EtLPP3-R (Primer for ORF sequences)	5'-CTACATGTGCGGCAGTTGAGG-3'
EtLPP2-F (Primer for ORF sequences)	5'-ATGGATGCTACCGATTCTGTAAC-3'
EtLPP2-R (Primer for ORF sequences)	5'-TCAATCCCTCAAATCGATGATG-3'
EtLPP1-F (Primer for ORF sequences)	5'-ATGGTTTCGCACGAGCCTG-3'
EtLPP1-R (Primer for ORF sequences)	5'-CTAACGACCATTTACATTCAAGACAAG-3'
EtLPP3-F (Real-time PCR primer)	5’- AGGCAACGACTGAGGAGCA -3’
EtLPP3-R (Real-time PCR primer)	5’- CGATAAAGAGGCAAGGAATGAGA -3’
EtLPP2-F (Real-time PCR primer)	5’-TCAGGCAATGATGTTGTTGGT-3’
EtLPP2-R (Real-time PCR primer)	5’- GATGAAGAAAGCCCCGAAAATAG-3’
EtLPP1-F (Real-time PCR primer)	5’- TCCCTTTCGTCCTCTCCTCA-3’
EtLPP1-R (Real-time PCR primer)	5’- CTGGCTTCGGCAACAATACA-3’
Et18S-F (Real-time PCR primer)	5’-TGGTGGAGTGATCTGTCTGGTT -3’
Et18S-R (Real-time PCR primer)	5’-CCTGTTATTGCCTCAAACTTCCTT -3’

### Transient expression of the His6-tagged *E*. *tenella* LPPs-encoded phosphatase in HEK-293 cells

The EtLPP plasmids were generated by insertion of the *E*. *tenella* LPPs sequence into the mammalian expression vector pcDNA3.1/His (Invitrogen, Grand Island, NY, USA). HEK 293 cells were plated into six-well dishes and grown to 50–60% confluence in 10% CO_2_. Transfection was performed using Lipofectamine, OPTI-MEM medium (Life Technologies, Gaithersburg, MD, USA), and 1–5μg of recombinant pcDNA3.1/His-expressing tagged EtLPP DNA per well. Control cells were transfected with the corresponding pcDNA3.1/His-vector DNAs. The DNA-containing medium was removed from the plate after 24h, and replaced by DMEM containing 10% FBS. After 48h, cells were washed and sonicated in lysis buffer [29]. The membrane fractions were prepared according to the method described previously [29], though with some modification (Ni-NTA agarose resin (Qiagen, Hilden, Germany) was used for protein immunoprecipitation). Protein concentration was determined by Bradford protein assay. SDS-PAGE and immunoblot analysis were conducted according to existing protocols [29].

### Enzyme Assays

Molecular Probes PiPer phosphate-release assay was used to measure the EtLPPs activity. This ultrasensitive assay detects free phosphate in solution through the production of the fluorescent product resorufin. Target protein was assayed for phosphatase activity against PA LPA, S1P, and C1P (Sigma-Aldrich Corporation, St. Louis, MO, USA) according to the supplier’s protocol. Preparation of substrate was conducted according to the method described previously [29, 30]. The reaction was started at 37°C by adding purified member protein (5μg) to a mixture (final assay volume 100μl) buffered with 0.01% phosphate-free triton X-100 (Sigma) and 1mg ml^-1^ fatty-acid free BSA (Sigma), pH 6.5, that contained substrate/Triton X-100 mixed micelles. Each reaction was performed in triplicate. The fluorescent product was detected using an Infinite M1000 instrument. The fluorescence value can be converted to the amount of organic phosphate present by comparison with a standard curve, made from the values obtained from the standard curve reactions. The protein purified from an empty vector was used as a control.

### mRNA expression pattern of PAPs in *E*. *tenella*

The PAP genes expression pattern during the parasite life cycle were analyzed by real-time quantitative RT-PCR (qRT-PCR) using the SYBR PrimeScript RT-PCR kit II (Takara Biotechnology, Dalian, China). Total RNA was extracted from unsporulated oocysts, differentially sporulated oocysts (1.5%, 48%, and 96%), sporozoites and merozoites, and further digested with DNase I (Takara, Japan) at 37°C for 15min. RNA quantities and qualities were determined by spectrophotometry and electrophoresis, judged by the presence of intact 28S and 18S rRNA bands at an intensity ratio of ~2:1. Real-time PCR was carried out in a final volume of 25μl containing 0.4μm of each primer (Table 1), 12.5μl of 2×SYBR Premix ExTaq and varied concentrations of cDNA templates. An Mx3000P real-time PCR machine (Agilent Stratagene, California, USA), with the recommended universal thermal cycling parameters, was used for amplification. Primers designed for these genes were synthesized by TaKaRa (Japan) and are shown in Table 1. 18S was used as an endogenous control. Each reaction contained at least three replicates. The expression of EtLPP1 in cDNA was used for calibration. A no-template control (NTC) was prepared using water as sample.

### Statistic analysis

Kinetic data were analyzed by Prism using a non-linear regression. One-way variance analysis (Prism) was used to determine statistical significance.

## Results

### Sequence analysis of the three EtLPPs

**Fig 1:**
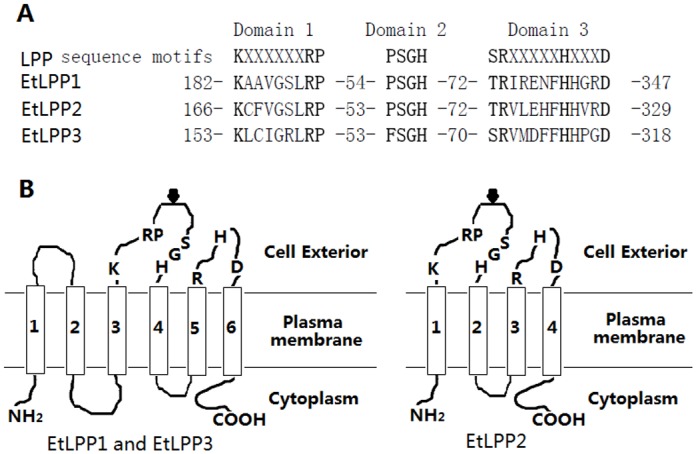
Sequence comparison of EtLPP1, -2, and -3. (A) the three conserved motifs in LPP family are shown on top of the alignment, three domains in deduced amino acid sequences of EtLPP1, -2 and -3 are listed. The predicted transmembrane topology of each of the three proteins is shown in B. (B) arrows represent glycolated sites. Those amino acids that are essential for activity within the three conserved domains are indicated by letters. Regions of hydrophobic amino acids are denoted 1 to 6, as predicted by Split 4.0.

Three cDNAs encoding LPP enzymes were found in *E*. *tenella* (KM880158, KM880159, and KM880160). The three LPP enzymes contained three conserved motifs, were designated as EtLPP1, -2 and -3, and consisted of 347, 329, and 318 amino acids (Fig 1A) with calculated *M*r values of 38239, 36483, and 34384, respectively. Amino acid sequences were distinct from each other. EtLPP1 was 41.5% identical to EtLPP2 and 28.9% identical to EtLPP3. EtLPP2 and EtLPP3 showed 27.2% identity. Additionally, comparison of the EtLPP sequences with three human LPP sequences disclosed low identical sequences except in a number of conserved regions. All three EtLPPs were shown to be glycosylated at a single consensus site (Fig 1B). EtLPP1 and EtLPP3 were predicted to have six transmembrane regions and EtLPP2 contained 4 putative transmembrane domains, according to hydropathy analysis (Fig 1B). Putative prosphatase-active sites were contained in three conserved domains and all of the domains were predicted to be in extracellular loops (Fig 1B). This structural prediction is similar with that of mouse LPP [12], suggesting that EtLPPs may act as LPP enzymes.

### Expression of the three EtLPPs in HEK293 cells

**Fig 2:**
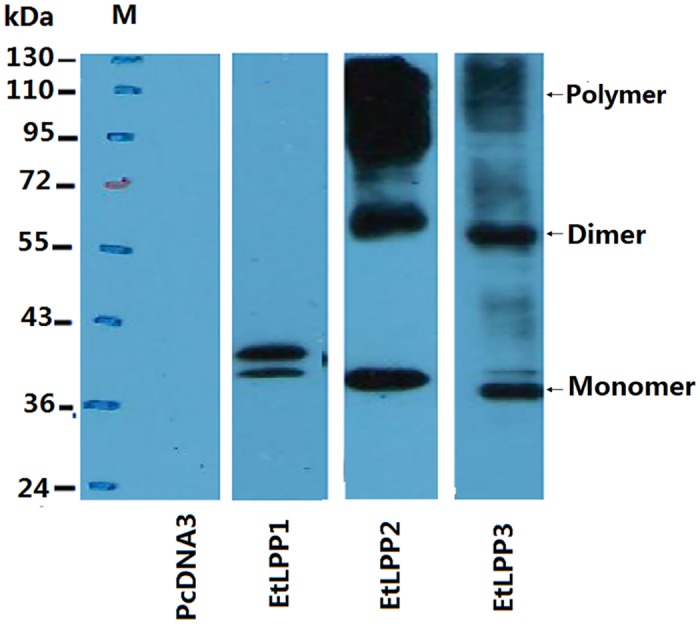
Western blotting confirming of the three EtLPP sizes. Expression of His6-tagged EtLPP1, -2, -3 or pcDNA3 in 293 cells was separated by SDS-PAGE (12%) and produced immunoreactive proteins by western blot using anti-His monoclonal antibody. Molecular weights of prestained markers are indicated in kDa.

The purified membrane proteins of the three His6-tagged EtLPPs were confirmed by immunoblot analysis using anti-His6 monoclonal antibody (Fig 2). In comparison with samples from empty-vector transfected cells, major immunoreactive proteins with estimated molecular masses of 40 and 42kDa; 40, 70, and 120kDa; and 37, 60 and 110kDa were detected in cells expressed by EtLPP1, -2 and -3, respectively (Fig 2). The immunoreactive bands of higher molecular weight (above 50kDa), observed in the case of EtLPP2 and EtLPP3, suggest the presence of multiple forms. Additionally, two immunoreactive bands below 50kDa were observed in each of the three expressed proteins (a minor immunoreactive band of EtLPP2 was occasionally observed and is not shown) (Fig 2), and probably represent the non-glycoslylated and glycosylated forms of EtLPPs.

### Enzymological properties of the three EtLPPs

**Fig 3:**
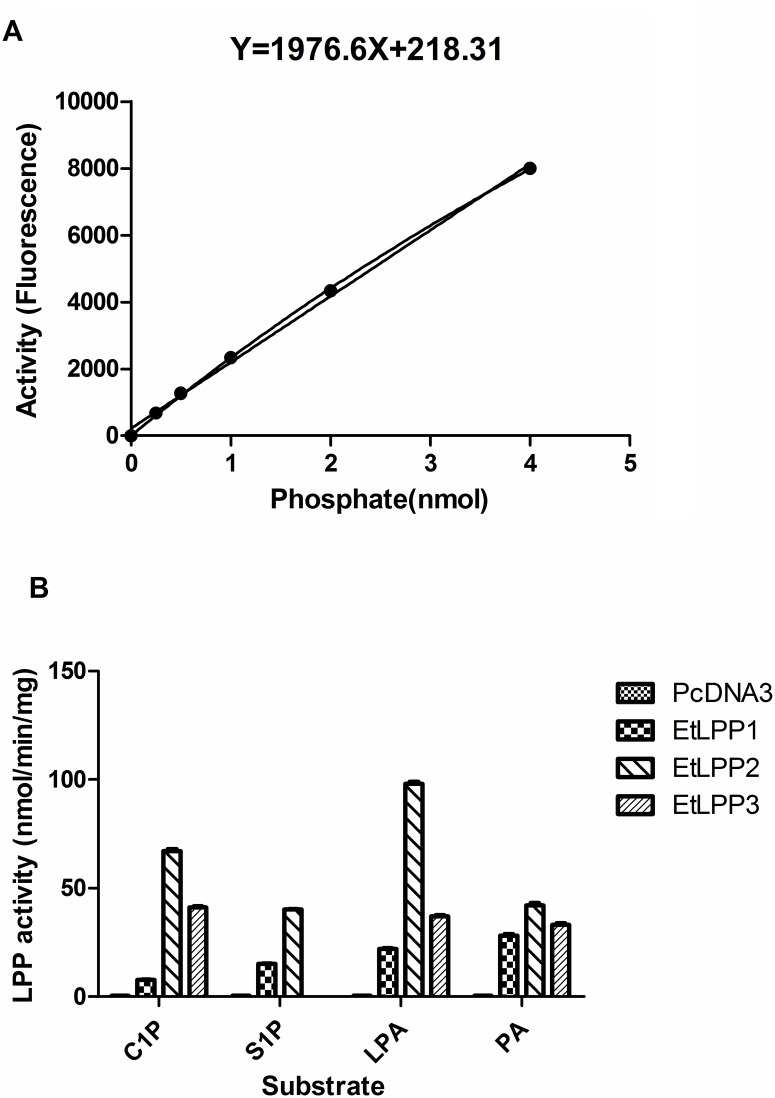
The LPP activity of each purified membrane protein is shown relative to the activity in vector transfected cells using PiPer phosphate-release assay. (A) Plotting phosphate standards against the corresponding fluorescent values. The data are means ± S.D. from the three samples for each standard. (B) The activities of the three EtLPPs were compared. The cDNAs encoding EtLPP1, -2 and -3 were sub-cloned in to pcDNA3 expression vector and transfected into 293 cells. Purified membrane proteins were used to the enzyme assay using PA, LPA, S1P and C1P as substrates. The values are means ± S.D. of three repeated experiments.

In order to confirm that the three immunopurified enzymes function as LPP enzymes, enzyme kinetics was performed. We used the Molecular Probes PiPer phosphate-release assay to detect the fluorescence of the fluorescent product resorufin. The increase in detectable fluorescence is proportional to the amount of phosphate present. As shown in Fig 3A, a line of best fit was used to calculate the amount of phosphate released from each sample. The amount of phosphate released from each protein sample is shown in Fig 3B. The activities of purified EtLPP1 to C1P, S1P, LPA and PA were 18-, 30-, 33- and 70-fold increase by comparison with the control, respectively. The activities of purified EtLPP2 to C1P, S1P, LPA and PA were 111-, 80-, 163-, and 105-fold increase, respectively. The activities of EtLPP3 against C1P, LPA, and PA were 68-, 61-, and 82-fold increases, respectively. However, EtLPP3 showed negligible activity on S1P (Fig 3B). This data revealed that the three cDNAs for the putative LPP enzymes (EtLPP-1, -2, and -3) encode functional LPPs in our assay system. LPP activity was not measured in the cytosol fractions of cells transfected with each of the three EtLPP plasmids (not shown). Additionally, the three EtLPPs were independent of Mg^2+^ and insensitive to N-ethylmaleimide (not shown), suggesting they are classified as Mg^2+^-independent and N-ethylmaleimide-insensitive plasma membrane-bound enzymes [31].

**Fig 4:**
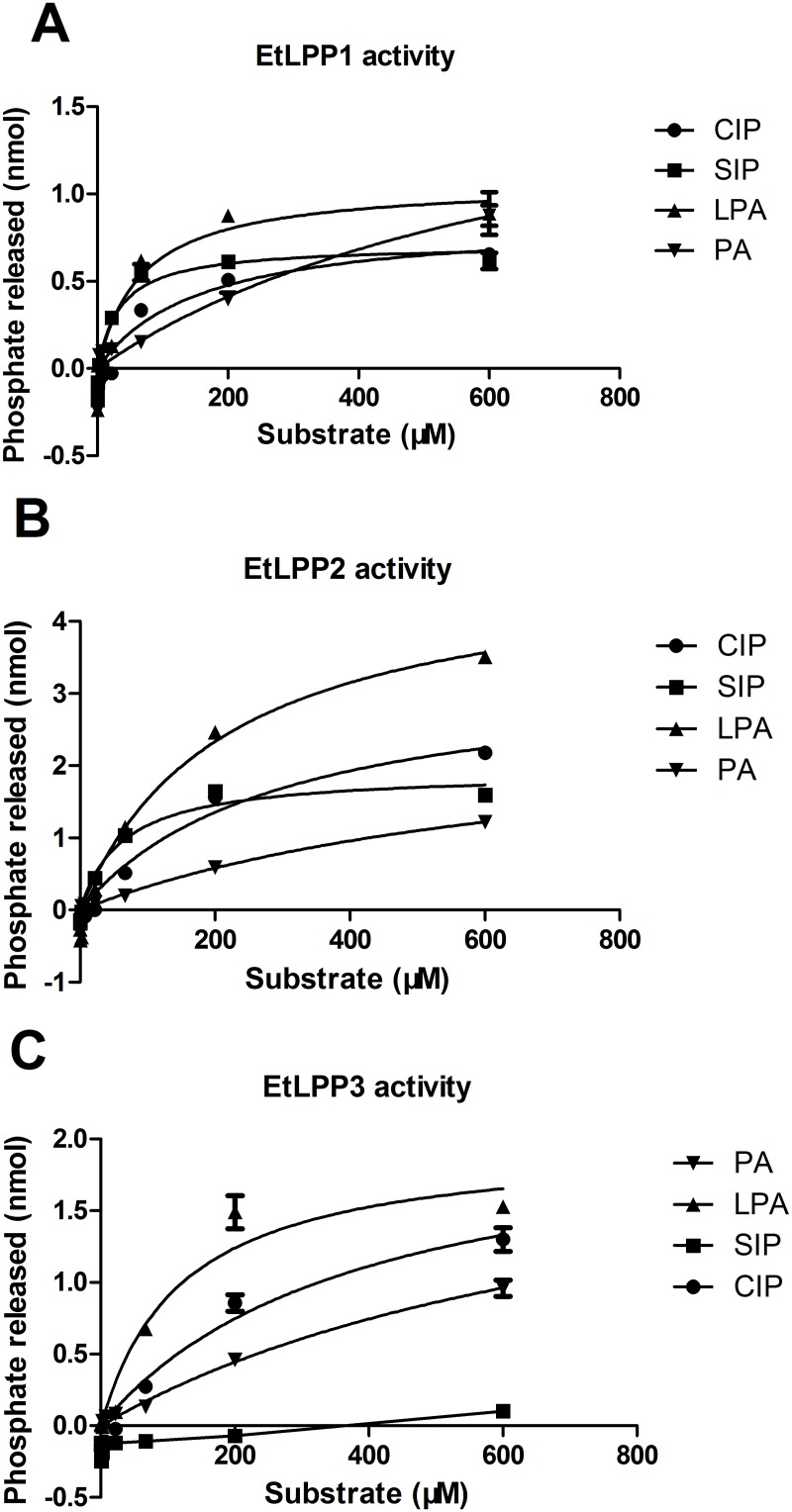
The kinetic analyses of each of the three EtLPP enzymes. The activities of each of the three EtLPPs were detected with C1P, S1P, LPA, and PA as substrates. Each concentration was assayed in triplicate. The data were analyzed using Prism Graphpad, using a non-linear regression.

Purified preparations of rat liver LPP have been reported to hydrolyze LPA, C1P, and S1P, in addition to PA [30]. Similarly, recombinant human PAP2a is highly active on PA and LPA, while PAP2b is active on PA and S1P. Thus, kinetic profiles of the three recombinant enzymes were developed using LPA, PA, S1P, and C1P as substrates to characterize their substrate specificity. The curves describing the kinetic profile of EtLPP-1, -2, and -3 using LPA, PA, C1P, and S1P as substrates are presented in Fig 4 and the derived *V*max and *K*m values are shown in Table 2. We also compared the relative ability of the different EtLPPs to dephosphorylate each substrate (Table 2). For EtLPP1, maximum activity was higher when PA was used as a substrate. For EtLPP2, maximum activity was higher when LPA was used as a substrate. EtLPP3 showed similar activity with PA, LPA, and C1P as substrates and was much less active with S1P as a substrate. Comparative *K*m values for the three recombinant expressed enzymes for each substrate showed that LPA, C1P, and S1P appeared to be selected substrates for the three EtLPPs, with distinct affinities. However, three recombinant-expressed enzymes had similar affinities to PA (*K*m~284–355μm).

**Table 2:** *K*m and *V*max values for the three EtLPP enzymes.

Substrate	EtLPP1		EtLPP2		EtLPP3	
	*V*max (nmol/min/mg)	*K*m (uM)	*V*max (nmol/min/mg)	*K*m (uM)	*V*max (nmol/min/mg)	*K*m (uM)
PA	0.28±0.08	284±54	0.42±0.01	369±72	0.33±0.07	355±61
LPA	0.22±0.03	46±9	0.98±0.01	179±35	0.37±0.07	83±11
C1P	0.08±0.02	28±5	0.67±0.01	237±47	0.41±0.05	260±51
S1P	0.15±0.01	22±6	0.40±0.04	52±11	0	0

### Expression profile of the EtLPPs

**Fig 5:**
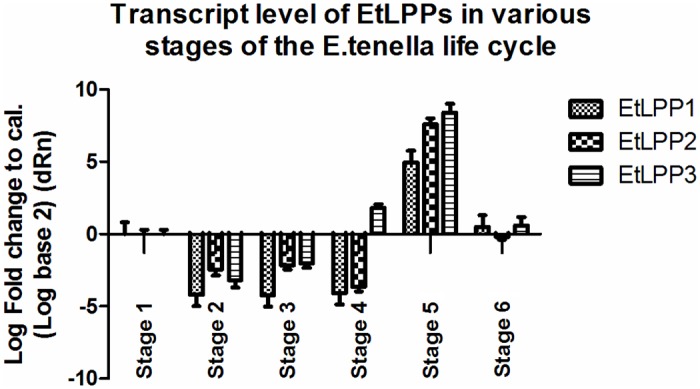
Expression profiles of the three EtLPPs in various stages of the *E*. *tenella* life cycle. Each of the EtLPP mRNA expression levels was measured based on three replicates using quantitative PCR. Stage 1, unsporulated oocyst; Stage 2, 1.5% sporulated oocyst; Stage 3, 48% sporulated oocyst; Stage 4, 96% sporulated oocyst; Stage 5, sporozoite; Stage 6, merozoite. The symbol (**) represents statistically different expression levels (one-way variance analysis; p<0.001).

We studied the relative levels of the three EtLPPs transcripts in unsporulated oocysts, differentially sporulated oocysts, sporozoites, and merozoites using quantitative real-time PCR (Fig 5). The presence of three EtLPPs was observed throughout all the investigated stages, suggesting the importance of regulating the balance between the lipid phosphates and their dephosphorylated products in the *E*. *tenella* life cycle. Conversely, we noted clearly divergent transcript levels in different cycle stages of *E*. *tenella* for all of the three EtLPPs. The three EtLPP transcripts were more alike in their significant upregulation of sporozoites (p<0.001), suggesting that their encoded proteins might play a pivotal role in lipid signaling during the sporozoite stage. Supporting this assumption is the fact that both mammalian and yeast LPPs are known to be involved in lipid signaling pathways [2].

## Discussion

In the present work, the three EtLPPs have been shown to have the LPP activities. The expressed EtLPP2 and EtLPP3 proteins showed variable molecular size, suggesting the presence of multiple forms of these proteins. However, EtLPP1 showed as a monomer. The two faster-migrating protein bands below each of the three EtLPP proteins were found to be very similar to the porcine 35-kDa LPP enzyme in their molecular size and the extent of N-glycosylation [32], suggesting that the three EtLPPs are partially N-glycosylated. The expressed EtLPP1 enzyme protein was found to exist as a monomer, suggesting the LPP activity measured with the immunoprecipitable protein in EtLPP1 plasmid-transfected cells can be accounted for only by an oligomer protein. The result is consistent with the observation that the porcine and mouse LPPs function as monomers with the 35-kDa sub-unit [32]. Additionally, EtLPP2 and EtLPP3 appeared in Fig 2 as monomer, dimmer and polymer forms. Actually, many works have reported that the native PAP shows a very large molecular weight [33, 34]. Siess et al. suggested that the rat LPP would contain a hexameric subunit structure [35]. Homooligomer may contribute to structural or functional stability for these LPPs. Smears were observed in Fig 2, probably caused by the presence of detergents in the enzyme preparation and prevented exact data interpretation. It would be interesting to investigate what forms of the three native EtLPPs exist in *E*. *tenella* and whether the three native EtLPPs function as a homooligomer or a monomer.

In this study, immunoprecipitated proteins from cells transfected with EtLPP1 or EtLPP2 gene were shown to have a broad substrate specificity and dephosphorylate a variety of lipid phosphates, including PA, LPA, C1P, and S1P. The data supplied a quantitative comparison of the relative activities of each of the three EtLPPs for the different substrates. The activities of expressed EtLPP enzymes were more similar to those of rat liver LPP, human, and yeast LPP enzymes [30], which are integral membrane proteins and have broad substrate specificity. These results reveal that the three EtLPP enzymes are like human and yeast LPP enzymes, having the ability to attenuate signaling by these lipids while simultaneously producing other signals through the formation of their counterparts. Measurements of the maximal rates and affinities of EtLPP1-3 by different substrate hydrolysis would provide useful information about their biological functions. It is worth mentioning that the three EtLPPs were found in *E*. *tenella*, all of which had LPP activity. We propose that the three EtLPPs may function redundantly. This assumption relies on past studies indicating that the LPP isoforms show functional redundancy in certain systems [36, 37]. Similarly, at least four different LPP isoforms have been found in humans [38, 39] and a large number of membrane-associated LPP-like proteins also have been found in bacteria, which have broad substrate specificity [40–44]. On the other hand, the reasons for the existence of different LPP enzymes are probably related to their different tissue and sub-cellular distribution and/or different substrate specificities. Thus, knowledge of the three EtLPP sub-cellular compartmentations may help to determine their different biological roles.

We succeeded in identifying the *E*. *tenella* LPP enzymes. Although we presented evidence of differences in the relative activity of the three EtLPPs on PA, LPA, C1P, and S1P, there are a number of questions to be addressed in future studies. Any further study needs to elucidate the physiological role of the three EtLPPs in the metabolism of these bioactive lipid substrates, define the exact sub-cellular localization for each EtLPP and determine whether marked higher-level transcription of the three EtLPPs during the sporozoite stage is related to biological functions of *E*. *tenella*. A better understanding of the activity and function of the LPPs in *E*. *tenella* will provide us with an exciting approach to inhibiting parasite development within chickens, where sporozoites are the key factor associated with poultry coccidiosis.
